# Acute pancreatitis mimicking hyperemesis gravidarum in the first trimester in young primigravida: diagnostic difficulty and a rare case report

**DOI:** 10.1097/MS9.0000000000005326

**Published:** 2026-07-20

**Authors:** Tanuja Khatri, Avi Pokhrel

**Affiliations:** Department of Obstetrics and Gynaecology, Manipal Teaching Hospital, Pokhara, Nepal

**Keywords:** acute pancreatitis, first trimester, hyperemesis gravidarum, rare case report

## Abstract

**Introduction and importance:**

Acute pancreatitis (AP) in pregnancy is rare, and its symptoms often overlap with common conditions like hyperemesis gravidarum (HG), which complicates diagnosis. Early recognition and appropriate management can prevent significant maternal and fetal complications.

**Case presentation:**

A 21-year-old primigravida at 10 + 6 weeks of gestation presented with the acute onset of abdominal pain in the epigastric region and multiple episodes of vomiting. She was provisionally diagnosed with HG, but further investigation revealed elevated serum lipase and amylase levels, leading to a diagnosis of AP. She was admitted to the intensive care unit for a multidisciplinary approach. The woman was finally discharged with stable vitals.

**Case discussion:**

In pregnant patients, severe HG symptoms can sometimes mask or co-occur with AP. Symptoms like severe abdominal pain and vomiting can overlap. Our patient presented with such symptoms, and it required thorough evaluation to reach our diagnosis and provide the appropriate care needed.

**Conclusion:**

This case highlights the importance of maintaining a high index of suspicion for pancreatitis in pregnant patients presenting with severe gastrointestinal symptoms, especially when the response to standard treatments for HG is suboptimal. Timely diagnosis and coordinated care are essential for optimizing outcomes.

## Introduction

Pancreatitis is a rare event in pregnancy, occurring in approximately 3 in 10 000 pregnancies. Although it is most often acute and related to gallstones, non-biliary causes should be sought because they are associated with worse outcomes^[^1^]^. Pancreatitis in pregnant women, especially acute pancreatitis (AP), is a challenge for both obstetricians and gastroenterologists^[^2^]^. Pancreatitis is more common with advancing gestational age^[^3^]^. Hyperemesis gravidarum (HG) is a condition causing severe nausea and vomiting in early pregnancy, often resulting in hospital admission^[^4^]^. HG typically begins between 4 and 6 weeks of gestation, affecting approximately 0.3–3% of pregnancies^[^5^]^. The most commonly cited criteria to define HG include persistent vomiting, acute dehydration and starvation (ketonuria), and weight loss >5%^[^6^]^. A 21-year-old primigravida at 10 weeks and 6 days of gestation presented with excessive vomiting – 10 to 12 episodes per day – and epigastric pain for 1 day. Basic investigations were done in the emergency room, and urine ketones were found positive. She was treated in line with HG. Despite optimal treatment for HG, the patient’s status did not improve. Further serum amylase and lipase tests were sent, yielding results 577 and 521 U/L, respectively. The case was then diagnosed as AP. The distinction between these two conditions can be challenging, especially in the first trimester when both can present with similar symptoms and result in significant morbidity if misdiagnosed. We present a case where AP was initially mistaken for HG. This case report has been prepared in line with the CARE guidelines^[^7^]^ and TITAN guidelines^[^8^]^.

HIGHLIGHTS

- Pancreatitis in pregnancy is rare, occurring in approximately 3 in 10 000 pregnancies. Acute pancreatitis (AP) is more common with advancing gestational age, making AP in the first trimester even rarer.
- The distinction between hyperemesis gravidarum and AP can be challenging, especially in the first trimester when both can present with similar symptoms and result in significant morbidity if misdiagnosed.
- Restriction of the use of various investigations, especially imaging modalities like computed tomography scans during pregnancy, further causes a diagnostic challenge.
- AP must be considered as a differential diagnosis for vomiting and abdominal pain in early pregnancy.
- Despite the diagnostic challenges involved, early recognition and appropriate management can prevent significant maternal and fetal complications.

## Case presentation

A 21-year-old female, primigravida at 10 + 6 weeks of gestation, presented to the emergency department with the chief complaint of abdominal pain for 1 day and vomiting 10–12 episodes on the day of presentation. She had a similar history 5 days ago and was managed as HG at another center. She had no other significant past history.

On examination, she was tachycardic. Abdominal examination revealed tenderness present in the epigastric region without guarding or rigidity. She was kept under a provisional diagnosis of HG in the ER and was managed with intravenous ranitidine 50 mg, hyoscine butyl bromide 10 mg, ondansetron 4 mg, and one pint of Ringer’s lactate. Baseline investigations, including renal function test, ultrasound (USG) obstetric scan, urine for ketones, urine routine examination, and random blood sugar, were performed.

**Figure 1.**
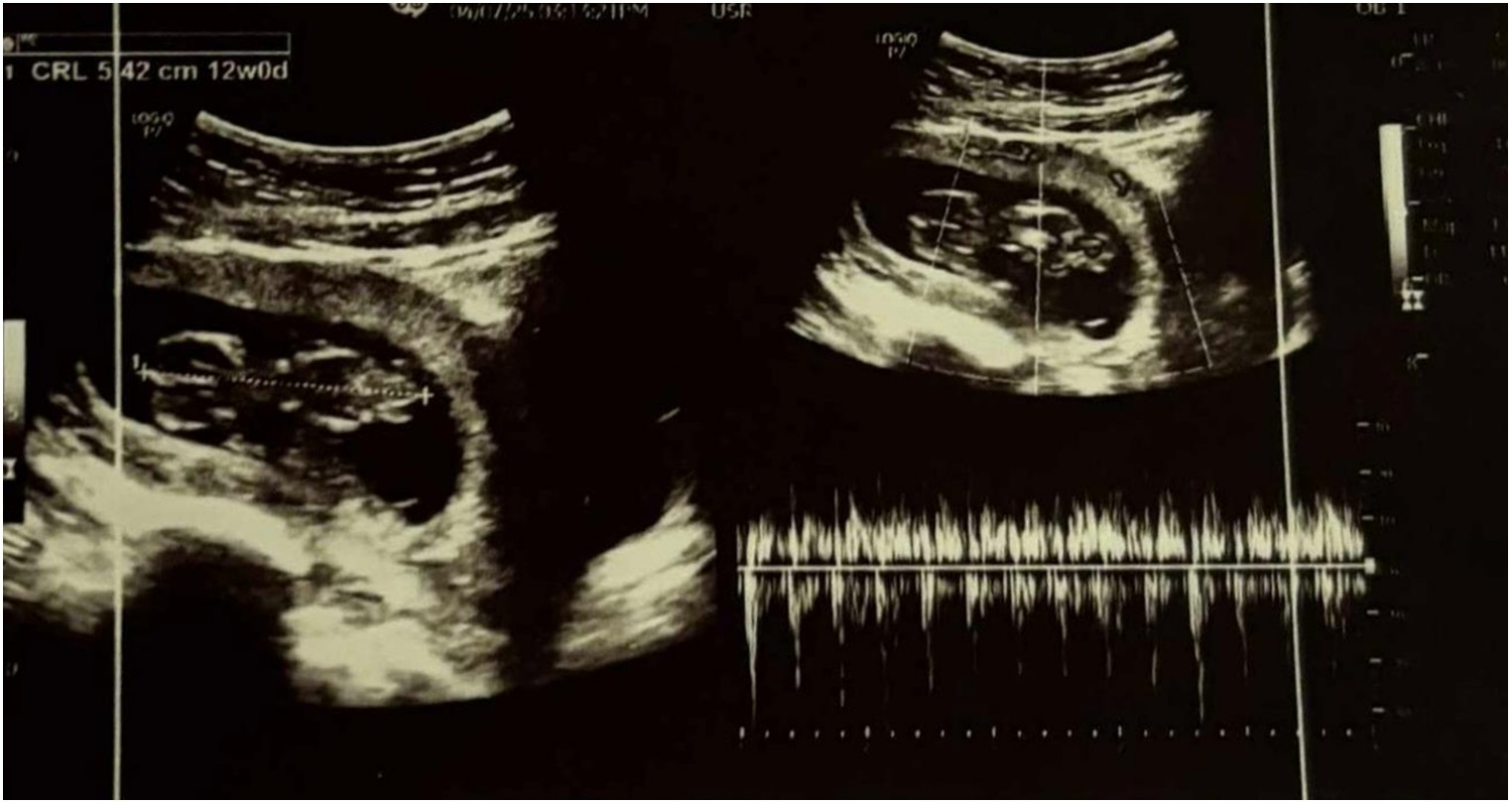
Obstetric ultrasound measuring crown–rump length (CRL).

The patient was subsequently admitted to the obstetric ward. Initial investigations revealed positive urinary ketones, while other parameters were within normal limits. On the second day of admission, her symptoms worsened, prompting further evaluation for AP. Serum amylase and lipase levels were measured and found to be elevated (577 and 521 U/L, respectively). Based on these findings, a diagnosis of AP was established. Following the reports and the worsening status of the patient, she was then shifted to the intensive care unit (ICU). Within 24 hours of admission, the BISAP score was calculated to be 0, indicating a low risk of mortality and severe complications from AP. The patient was kept nil per os and managed with intravenous fluids (Ringer’s lactate, dextrose normal saline, and 10% dextrose with potassium chloride supplementation), along with analgesics (intravenous paracetamol 1 g), antiemetics (intravenous ondansetron 4 mg), and proton pump inhibitor therapy (intravenous pantoprazole 40 mg). USG abdomen and pelvis, serum calcium, and lipid profile were performed in the ICU to gather further information on the cause and severity of AP. Her serum calcium was 9.4 mmol/L, and the lipid profile showed triglycerides 102 mg/dL, low density lipoprotein 96 mg/dL, and total cholesterol 157 mg/dL, all within normal limits. Transabdominal ultrasonography revealed a single live intrauterine pregnancy with a fetal pole (Fig. 1), corresponding to 12 weeks of gestation based on crown–rump length. Serum IgG4 levels were measured to rule out autoimmune pancreatitis and were within normal limits.

The initial laboratory parameters of the patient upon presentation at the tertiary center are given in Table 1.

**Table 1:** Laboratory parameters.

Laboratory parameters	Patient value	Ref. range
LDH	466 U/L	313–618 U/L
Lipase	**521 U/L**	12–53 U/L
Amylase	**577 U/L**	30–118 U/L
Lipid profile		
Triglyceride	102 mg/dL	50–150 mg/dL
Cholesterol	157 mg/dL	150–200 mg/dL
HDL cholesterol	40.7 mg/dL	40–60 mg/dL
LDL cholesterol	96 mg/dL	<100 mg/dL
Hematology		
Total leukocyte count	10 300/cu.mm	4000–11 000/cu.mm
Hb	12.7 gm%	13–17 gm%
PCV	33.8%	40–50%
RBC	4.10 million/cu.mm	3.5–4.5 million/cu.mm
Platelets	356 000/cu.mm	150 000–450 000/cu.mm
Biochemistry		
Glucose random	98 mg/dL	70–140 mmol/L
Urea	23 mg/dL	10–45 mg/dL
Creatinine	0.51 mg/dL	0.6–1.2 mg/dL
Sodium	140 mEq/L	135–145 mEq/L
Potassium	4.11 mEq/L	3.5–5.1 mEq/L
Total bilirubin	0.6 mg/dL	0.2–1.3 mg/dL
SGPT/ALT	25 U/L	0–35 U/L
SGOT/AST	21 U/L	14–36 U/L
Albumin	**5.2 g/dL**	3.5–5.0 g/dL
Alkaline phosphatase	54 U/L	38–126 U/L
Serum total calcium	9.4 mg/dL	8.3–10.6 mg/dL
Parathyroid hormone	30.48 pg/mL	15–65 pg/mL
Urine for ketones	**Positive**	
Immunoglobulin subclass		
IgG-4	0.18 g/L	0.1–1.2 g/L

AST, aspartate aminotransferase; HDL, high density lipoprotein; PCV, packed cell volume; RBC, red blood cell; SGOT, serum glutamic oxaloacetic transaminase; SGPT, serum glutamate pyruvate transaminase. Bold values signifies abnormal labaratory findings in the patient.

She was managed conservatively in the ICU for 5 days. Her symptoms gradually improved, and she was shifted to the obstetrics ward. After being shifted, she was kept on a tolerable diet and was finally discharged with stable vitals.

## Discussion

The differential diagnosis of epigastric pain in the first trimester includes HG, biliary tract disease, peptic ulcer disease, gastritis, and hepatic disorders. Although HG is frequently encountered, it may obscure less common but clinically significant conditions such as AP. In this case, ultrasonography did not demonstrate gallstones or biliary pathology, and there were no clinical or laboratory features suggestive of peptic or hepatic disease. The diagnosis of AP was established based on elevated pancreatic enzymes and supportive imaging findings. A BISAP score of 0 further indicated a mild disease course.

The diagnosis of AP is based on the Revised Atlanta Classification, which requires the presence of at least two of the following criteria:

- Characteristic abdominal pain, defined as epigastric discomfort that often radiates to the back.
- Serum amylase or lipase level three or more times the upper limit of normal.
- Imaging findings consistent with AP on ultrasound, computed tomography (CT), or magnetic resonance imaging scans.

Clinical presentation and elevated pancreatic enzymes are usually sufficient to establish the diagnosis. Imaging is typically reserved for patients with diagnostic uncertainty or a clinical course that is severe or worsening^[^9^]^.

In published series on acute pancreatitis in pregnancy (APIP), abdominal pain is the most frequently reported symptom (86.8%), followed by vomiting (73.6%)^[^10^]^.

The most common causes of APIP are gallstones (66%), alcohol abuse (12%), idiopathic (17%), hyperlipidemia (4%), and, less commonly, hyperparathyroidism, trauma, medication, and fatty liver of pregnancy^[^11^]^.

In this case, there was no history of alcohol use or intake of medications known to be associated with AP. Serum triglyceride and lipid levels were within normal limits, serum IgG4 was negative, and abdominal ultrasonography did not demonstrate gallstones or biliary tract disease. Based on the absence of common etiological factors, the pancreatitis in this patient was most likely idiopathic.

In pregnant patients, severe HG symptoms can sometimes mask or co-occur with AP. Symptoms like severe abdominal pain and vomiting can overlap. Investigations to diagnose AP include blood tests and imaging. Abdominal ultrasound, CT scan, endoscopic ultrasound, and magnetic resonance cholangiopancreatography are the available imaging studies for diagnosing a biliary etiology for AP. Potential radiation to the fetus is a major disadvantage with CT scans, substantially restricting their use^[^12^]^, thus further highlighting the diagnostic challenges involved.

HG is managed with antiemetics and IV fluids, but AP, which carries significant maternal and fetal morbidity and mortality risks, necessitates accurate and timely diagnosis and different treatments, including nil per oral, and may necessitate more urgent intervention depending on the cause, making it essential to run tests to differentiate between the two conditions.

## Conclusion

AP in pregnancy is a rare entity, most commonly reported in the second and third trimesters, while first-trimester presentations are uncommon and may closely mimic HG, leading to a diagnostic delay. This case highlights the importance of considering AP in pregnant patients presenting with persistent vomiting and epigastric pain, particularly when there is a poor response to standard management for HG. Early biochemical evaluation with serum amylase and lipase is essential to avoid misdiagnosis, especially in the presence of overlapping findings such as urine ketone positivity, which may reflect starvation and is not specific to HG alone. In this case, a comprehensive etiological workup, including gallstones, hypertriglyceridemia, hypercalcemia, and autoimmune pancreatitis, was negative, suggesting an idiopathic etiology.

This case also underscores how initial diagnostic framing as HG, combined with ketonuria and early pregnancy presentation, may introduce cognitive bias and delay appropriate investigation. Clinicians should maintain a high index of suspicion and consider pancreatic enzyme testing in any pregnant patient with persistent symptoms beyond 24–48 hours despite standard therapy.

## Data Availability

The data supporting the findings of this case report are available from the corresponding author upon reasonable request. All relevant data are included within the manuscript, and patient confidentiality has been maintained in accordance with ethical standards.

## Research registration unique identifying number (UIN)

ResearchRegistry11672.

## Patient perspective

Patient was initially anxious due to her worsening symptoms and ICU admission. At one point, she expressed a desire to terminate the pregnancy because of fear for her own health. However, as her condition gradually improved, she felt reassured and chose to continue the pregnancy. At discharge, she reported feeling relieved, emotionally supported, and optimistic, and she expressed satisfaction with the outcome of her care.
